# Prenatal Exposure to BPA and Sexually Selected Traits in Male Mice

**DOI:** 10.1289/ehp.119-a383

**Published:** 2011-09-01

**Authors:** Wendee Holtcamp

**Affiliations:** Wendee Holtcamp, an award-winning writer based in Houston, TX, has published in *Nature*, *Scientific American*, *BioScience*, *Climate Central*, and other publications.

Bisphenol A (BPA), an industrial chemical used to manufacture epoxy resins, polycarbonate plastics, and thermal paper, has come under fire because of accumulating evidence of its harmful effects on test animals and, potentially humans.^1^ A new mouse study now suggests BPA may adversely affect development of sexually selected traits, potentially compromising animals’ ability to reproduce.^2^

Sexually selected traits are those that make individuals more attractive to potential mates, enhancing their reproductive success. Sometimes these traits come at a cost—for instance, elongated tail feathers in some male birds attract not only more females but also more parasites.^3^ How sexually selected traits manifest in a given individual strongly depends on outside factors, such as maternal condition during gestation or other factors in the environment as an individual matures.^4^ Some scientists hypothesize that endocrine-disrupting chemicals also could influence these traits.

The new study was supported by an American Recovery and Reinvestment Act Challenge Grant from the National Institute of Environmental Health Sciences to study health effects of BPA.^5^ Grantee Cheryl Rosenfeld, an associate professor of biomedical sciences at the University of Missouri, along with psychology professor David Geary and graduate student Eldin Jašarević, used evolutionary ecology theory to help design the study. “We focused on spatial cognition because the hippocampus, the brain region that affects this trait, is evolutionarily conserved between rodents and humans,” Rosenfeld explains.

The investigators selected deer mice as an animal model because males in this species depend upon enhanced navigational skills—a sexually selected trait—to seek out mates during the breeding season. (Females, in contrast, tend to stay close to the nest and do not exhibit enhanced navigation abilities.) Comparable traits in humans might lead men in traditional societies to travel farther than women to form political alliances, for raiding, warfare, courting, and hunting; in general, men tend to possess better spatial navigational abilities than women.^6^^,^^7^

The study assessed whether dietary exposure to BPA in pregnant and nursing deer mice affected spatial navigational abilities in offspring that were exposed to the chemical only through their mother. During pregnancy and nursing, adult females were fed 50 mg BPA/kg feed, a dose believed to yield blood concentrations of BPA^8^ comparable to those reported in the general human population.^9^ After weaning, all offspring were placed on a normal diet.

Sexually mature male offspring exposed to BPA during development showed neither obvious physical abnormalities nor reduced sensory or neuromuscular abilities (which are not sexually selected traits). However, compared with control males and females, exposed males showed reduced ability to use memory and spatial cues to navigate a Barnes maze, a circular apparatus with 1 exit hole and 11 false exits. Likewise, exposed males performed worse in an elevated-plus maze, a device with open and closed arms, which tests exploratory behavior and anxiety-like behaviors. “More time in the closed arms equates to increased anxiety-like behaviors and decreased willingness or confidence to explore and seek out mates,” Rosenfeld says.

Two weeks after the navigational tests, the investigators conducted a mate choice experiment. They measured interest in mating as time spent by prospective mates in nose-to-nose contact. Sexually mature females showed two times less interest in mating with BPA-exposed males than they did with control males. The scientists were not able to determine whether reduced sexual interest was due to behavioral cues, chemical signaling, or both.

How might BPA affect these reproductive traits during development? Rosenfeld suspects the mechanism is epigenetic, in which the chemical does not alter the DNA itself, just its ability to be transcribed and eventually translated into proteins.^10^ However, the investigators can’t rule out impacts of maternal care on the offspring given that the dams were directly exposed to BPA.

“These data provide an important new result in a species that is far closer to a wild mouse,” says Pat Hunt, a professor of molecular biosciences who first accidentally discovered that BPA damaged mouse ova when a strong detergent containing the compound was used to clean cages.^11^ “The obvious question is: Does this mean that it could affect gender identity in humans? Admittedly, that is quite a step away, but it does suggest that, at the very least, BPA exposure might make men less ‘manly.’”

Similarly, Geary says the study has implications for traits that should be more closely examined in humans to detect potential BPA impacts. “Now we can say, given [these results], if we’re going to assess risk in boys we should look at complex spatial learning, rough-and-tumble play, and interest in object play,” he says. Geary lists these as human sexually selected behaviors in his book *Male, Female: The Evolution of Human Sex Differences.*^12^

Related studies are beginning to emerge. In 2009 Harvard University School of Public Health research fellow Joe Braun and colleagues reported that girls exposed prenatally to BPA were more likely than exposed boys to exhibit hyperactivity and aggression at age 2 years.^13^ Geary points out, though, that it’s impossible to know whether aggression at age 2 is related to dominance, which is a sexually selected trait (among boys), or to characteristic toddler impulsivity, which is not. He says, “I would feel more comfortable attributing the results to sexual selection if [the investigators] can show that these girls are more dominant, socially, as they get older.”
